# Relationship Between Perceived Social Self-Efficacy and Depression in Adolescents

**Published:** 2014

**Authors:** Zaeema Riaz Ahmad, Saba Yasien, Riaz Ahmad

**Affiliations:** 1 Associate Professor, Institute of Clinical Psychology, University of Karachi*, *Sindh, Pakistan.; 2 Institute of Clinical Psychology, University of Karachi, Sindh, Pakistan.; 3 Director and Associate Professor, Institute of Clinical Psychology, University of Karachi, Sindh, Pakistan

**Keywords:** Adolescents, Depression, Perceived Social Self-Efficacy, Siddiqui-Shah Depression Scale

## Abstract

**Objective:** The objective of the present study is to investigate the relationship of perceived social self-efficacy (PSSE) with depression in students.

**Methods:** The sample of the present study consisted of 216 adolescent students including 120 males and 96 females randomly selected from different educational institutes of Karachi, Pakistan. The age of the participants ranged from 16 to 19 years old, with the mean age of 17.09 ± 1.13 years. Personal information form, PSSE scale, and Siddiqui-Shah depression scale were administered on the adolescent students to check the following hypothesis; there would be a negative correlation between PSSE and depression in adolescents.

**Results: **Pearson product moment coefficient of correlation was applied to analyze the relationship between PSSE and level of depression in adolescent students. Findings of the study showed a significant negative correlation (r = −0.149, p < 0.05) between the variables of PSSE and depression.

**Conclusion:** There is a relationship between PSSE and depression in adolescents.

## Introduction

The sense of self plays a vital role in human beings’ psychological well-being, physical health, and interpersonal relationships. Bandura (1) described that individuals have a self-system with the capabilities to control over their thoughts, feelings, and behaviors, “What people think, believe, and feel it effects how they behave”. This conceptual framework emphasizes that cognition plays an important role in people’s capability to perceive the situation, to execute the behaviors and construct the reality.

The core of social cognitive theory and human agency is self-efficacy beliefs that strongly affect the human functioning. According to Graham and Weiner (2), perceived self-efficacy is a more consistent predictor of behavioral outcomes than any other motivational construct. Perceived self-efficacy refers to “beliefs in one’s capabilities to organize and execute the courses of action required managing prospective situations (3). The implication of self-efficacy in the social domain is known as perceived social self-efficacy (PSSE). PSSE is defined as an individual’s confidence in her/his ability to engage in social interactional tasks necessary to initiate and maintain interpersonal relationships (4).

This level of social confidence helps to play an active role in every area of life. These self-efficacy beliefs in a social context have the power to improve performance in academic tasks (5, 6), career choice (7), to generate optimism (8) that eventually diminish the thoughts of hopelessness which is the prominent symptom of depression, and reduces the level of depression (9). 

Self-efficacy beliefs regulate human functioning through cognitive, motivational, affective, and selection processes (1, 3, 10). The options, aims, effort to achieve these aims or goals, and persistence of efforts by an individual can be impacted by individual’s self-efficacy (11, 12). Transitional changes can challenge and possibly destabilize competency related belief. Competency in the social setting and emotional stability form a critical foundation for academic achievement and is considered as a protective factor against psychopathologies. Sun (13) described competency in social situations as “aspects of social behavior that are important with respect to preventing physical illness or psychopathology in children and adults”. Self-efficacy beliefs influence individual’s thought patterns and emotional reactions in a way that positive mood enhances perceived self-efficacy and despondent mood diminishes it (14, 15).

Depression is a widespread disorder specifically among adolescents, which is alarming for mental health professionals. Depression is one of the disorders, which can develop in any period of life and affect the cognitive, affective and behavioral aspects of life. Depression is a mood state characterized by lowered self-esteem, lack of interest in usual activities, weight loss or gain, insomnia or hypersomnia, psychomotor agitation or retardation, fatigue, diminished ability to think or concentrate, indecisiveness, and suicidal ideation or attempt (16).

Childhood depression is widely recognized, and health professionals see depression as a serious and major mental health problem in both adolescents and young children (17). Based on comprehensive local clinic based studies in all provinces of Pakistan, the findings regarding the prevalence of depression were: Sindh: 16% urban, 12% rural, Punjab: 8% urban, 9% rural, Baluchistan: 40% urban, 2.5% rural, NWFP: 5% urban, 3% rural (18). Interpersonal problems have found to be one of the causes of depression (19). Depressive symptoms were also found to be the one of the strongest predictor of suicidal ideation (20). In USA, suicide was the third leading cause of death among youth and young adults aged 10-24 years (21). Khokher and Khan (22) found the overall rate of suicidal ideation as 31.4% in Pakistani college students. 

Depression may be created by cognitive distortions, stressful life events, and physiological states. Regarding cognitive aspect, a person become depressed when perceives himself unable to control the valued outcomes (12). Hoffman et al. (23) highlighted the role of faulty and negative self-perceptions in the maintenance and causes of depression. Bandura (14) confirmed that human depression is cognitively caused by dejected ruminative thoughts and also low sense of efficacy to control over these ruminative thoughts contributes to the development of depression. Another study found the relationship between negative self-statements and self-efficacy, and both general self-efficacy and social self-efficacy play a mediating role between negative self-statements and social anxiety (24). Poor social integration has also been found as one of the factors that causes the onset (25) and course of depressive symptoms (26, 27).

These cognitive models of depression draw the attention of mental health professionals to identify the cognitive errors leading towards depression and also to make the interventions that correct these errors. A large number of researches have proved that individuals who suffer from depression reported poor self-efficacy, low self-esteem, feelings of worthlessness, an external locus of control, and experience guilt or shame over their limitations (28-31). As physiological, cognitive and social changes are on its peak in youth, it raises the need to structure the environment in a way that develop or enhance the positive cognitions in adolescents. These findings suggest that if people enhance their efficacious beliefs in social situations, they can avoid from falling into depression. As a result, it is very important to check this phenomenon and to change thought processes in a positive way particularly beliefs about their capabilities or competency.

In Pakistan, few researches have been conducted to explore the role of self-efficacy, for example, self-efficacy and mathematics achievement (32), self-efficacy and depression in physically handicapped children (33), and teacher stress, job performance and self-efficacy of women (34). However, these researches are not sufficient in this area and demands further extensive studies on this variable. Therefore, the present study is an attempt to open newer avenues of understanding the concept of self-efficacy and its relationship with psychological well-being of adolescents in Pakistani society.

## Materials and Methods

***Sample***

The entire sample for the present study consisted of 216 adolescent students with the age range of 16-19 years (mean age: 17.09 years). The respondents were selected from the educational institutes registered under Ministry of Education province of Sindh. The sample was again divided into low, middle, and high socioeconomic classes on the basis of house hold income and expenditure survey conducted by the Federal Bureau of Statistic, Government of Pakistan (35). Individuals from each socioeconomic class were included.

***Material***

*Personal information form*

Personal information was obtained through items focusing on the participant’s age, gender, birth order, number of siblings, education, etc. Educational organization related information was obtained through items which focus on the name of organization and type that is, private and government. Parent related information was based on parent’s marital status, parental education, employment status, and nature of the job.

*PSSE (Urdu version)*

PSSE scale (original version) was developed by Smith and Betz (4). It consists of 25 rationally derived items that measure the level of confidence in a variety of social situations. Responses are obtained using a five-point Likert scale ranging from 1 (no confidence at all) to 5 (complete confidence).

Smith and Betz (4) reported Cronbach’s alpha value of 0.95, an internal consistency reliability coefficient of 0.94 (N = 354) for the scale and conducted test-retest reliability in a smaller sample (N = 109) over an interval of 3 weeks, yielding a value of r = 0.82, demonstrating evidence of reliability. Concurrent validity data were obtained using the social subscale of the self-efficacy scale (36) and the social confidence scale of the skills confidence inventory (37). Correlations of these scales with the PSSE were r = 0.60 for males and r = 0.62 for females on the former, and r = 0.46 for males and r = 0.53 for females for the latter. This demonstrates that the instrument is sufficiently similar to a scale designed to measure the same construct as well as one designed to measure the construct of social confidence in the career domain. Significant concurrent construct validity evidence was also found by comparing the measure with the similar variables of social anxiety and shyness (4). Correlations of this measure with a measure of shyness were r = −0.67 for males and r = −0.71 for females. For social anxiety, correlations of r = −0.57 for males and r = −0.68 were found. PSSE scale was developed by Smith and Betz (4). 

Adapted version of scale (38) consists of 23 items that measure the level of confidence in a variety of social situations. Cronbach’s alpha value of adapted version is 0.902. Test-retest reliability in a smaller sample (N = 40) over an interval of 1 week yielding a value of r = 0.887, and split-half reliability was 0.797 demonstrating evidence of reliability. Significant convergent validity evidence was also found by comparing the measure with the Rosenberg self-esteem scale (39) r = 0.476 and generalized self-efficacy scale (40), r = 0.512 on a sample of (N = 67).

*Siddiqui-Shah depression scale (SSDE)*

Siddiqui-Shah depression scale (SSDE) is a self-report scale to measure depression in both clinical and non-clinical Pakistani populations. Each item is rated on 4-point rating scale from 0 to 3 (0 = never to 3 = most of the time). The total score is obtained by summing scores on all items. The scores range is 0-108. Low scores indicate absence or lower levels of depression and high scores indicate higher levels of depression. The split-half reliabilities of the scale with Spearman-Brown correction were r = 0. 79 and r = 0.84 for the clinical and r = 0.80 and r = 0.89 for the non-clinical samples, respectively. Alpha coefficients for the clinical and non-clinical samples were 0.91 and 0.89, respectively. The scale correlated significantly with Zung’s depression dcale, r = 0.55 (p < 0.001) and psychiatrists’ ratings of depression r = 0.40 (p < 0.05). 

***Design and procedure***

This is a correlational study conducted in the year 2010. All the procedures followed, and material used were reviewed and approved by the Departmental Research Committee, Institute of Clinical Psychology, University of Karachi. The authorities of the educational organizations were approached to gain permission to conduct the study on adolescent students. Students were informed about the benefits of the study and consent form was introduced. Each of the participants who voluntarily decided to participate in the study signed the consent form. Through that the consent form, students were informed that information gathered would be confidential and that their refusal to participation and right to withdraw from the study would not affect grades or status in College/University. 

Personal information form, PSSE scale, Urdu version (38) and SSDS (41) were administered in the group setting respectively with 5 min breaks. Not more than 10 adolescent in the group were taken for administration at a time. After data collection, the answer sheets were scored according to the standard procedures. Pearson product moment coefficient of correlation was applied to analyze the relationship between PSSE and level of depression in adolescent students.

## Results

Descriptive statistics of frequencies, percentages, mean, and SD were calculated for the demographic variables of gender, age, education, and income group (Table 1). The age range of the students was from 16 to 19 years old with the mean age of 17.09 (±1.13), and 55.6% of the sample consists of males with the mean age of 17.29 (±1.14) and 44.4% consists of females with the mean age of 17.01 (±1.12). About 28.7% of the sample were students of 10^th^ grade and 71.3% were students of 12^th^ grade. Further, 37.5% belong to lower middle class socioeconomic status, 37.5% of the sample belongs to middle class socioeconomic status, and 25.0% of the sample belongs to upper middle class socioeconomic status.

**Table 1:** Descriptive statistics of demographic variables

**Variables**	**N**	**%**
**Gender**		
**Male**	120	55.6
**Female**	96	44.4
**Age (years)**		
**16**	58	24.9
**17**	39	16.7
**18**	17	7.3
**19**	27	11.6
**Mean ± SD age for total sample**	17.09 ± 1.13	
**Mean ± SD age for males**	17.29 ± 1.14	
**Mean age ± SD for females**	17.01 ± 1.12	
**Education**		
**10** ^th^ ** grade**	62	28.7
**12** ^th^ ** grade**	154	71.3
**Income group**		
**Lower**	81	37.5
**Middle**	81	37.5
**Upper**	54	25.0

Mean scores of SSDS and PSSE scale in the total study population and each gender and age group are presented in tables 2-4. Results show that PSSE displayed significant negative correlations in the expected direction. The more strongly adolescents endorsed the beliefs of self-efficacy in social situations less depression they experienced (r = −0.149, p < 0.05). Results are presented in table 2.

**Table 2:** Correlation between perceived social self-efficacy and Siddiqui-Shah depression scale

**Scales**	**M**	**R**	**Sig.**
**PSSE** †	78.96	−0.149	0.05
**SSDE** ‡	27.21		

† Perceived social self-efficacy;

‡ Siddiqui-Shah depression scale

Correlation between PSSE and depression in male and females was also explored. As table 3 shows self-efficacy beliefs in social situation were negatively correlated with depression in males (r= −0.130, p < 0.20) and females (r = −0.114, p < 0.21).

**Table 3:** Correlation between perceived social self-efficacy and Siddiqui-Shah depression scale in males and females

**Gender**	**Scales**	**M**	**r**	**Sig.**
**Males**	PSSE†	81.08	−0.130	0.20
	SSDS‡	24.67		
**Females**	PSSE	76.36	−0.114	0.21
	SSDS	30.65		

† Perceived social self-efficacy;

‡ Siddiqui-Shah depression scale

In regard to correlation coefficients of PSSE and depression in each gender showed that higher score on PSSE scale were negatively correlated to symptoms of depression in each age group including 16 years old (r = −0.080, p < 0.56), 17 years old (r = −0.121, p < 0.51), 18 years old (r = −0.455, p < 0.10) and 19years old (r= −0.389, p < 0.38) although this is statistically weak relationship as presented in table 4.

**Table 4:** Correlation between perceived social self-efficacy and Siddiqui-Shah Depression Scale in each age group

**Ages**	**Scales**	**M**	**R**	**Sig.**
**16 years**	PSSE†	79.72	−0.080	0.568
	SSDS‡	24.15		
**17 years**	PSSE	82.10	−0.121	0.516
	SSDS	27.94		
**18 years**	PSSE	79.00	−0.455	0.102
	SSDS	19.14		
**19 years**	PSSE	84.50	−0.204	0.389
	SSDS	24.65		

† Perceived social self-efficacy;

‡ Siddiqui-Shah depression scale

## Discussion

The objective of the present study was to investigate the correlation between PSSE and depression in the developmental period of late adolescence. Analysis of the result shown in table 2 (PSSE M= 78.96, SSDS M = 27.21, r = −0.149, p < 0.05) indicate that there is a negative relationship between level of depression and PSSE in adolescent students.

Findings of the present study are consistent with the formulated hypothesis and with previous studies. A study conducted by Bandura and colleagues (42), found not only the relationship of social self-efficacy to depressive symptomatology, but it may also influence a variety of outcomes as prosocial behavior and academic achievement. Stroiney (43, 44) have also found the relationship and mediational role of PSSE with depression. In addition, social self-efficacy, self-esteem, and gender role variables may serve to protect against depression and loneliness. Kim and Cicchetti (45) also proposed that social self-efficacy serves as a protective factor in the link between maltreatment and internalizing symptomatology. Smith and Betz (46), relates these construct to career decision efficacy, establishes a path between social self-efficacy and shyness, and a continuation of the path to depression. 

In addition, table 3 illustrates negative correlation between scores of PSSE and depression in males (r = −0.130, p = 0.20) and females (r = −0.114, p = 0.21). Scores of the PSSE scale and depression shows a negative correlation in each age group as r = −0.080, p = 0.568 in adolescents of 16 years of age, r = −0.121, p = 0.516 in 17 years, r = −0.455, p = 0.102 in 18 years and −0.204, p = 0.389 in 19 years old college students presented in table 4. On the basis of such results, we consider that a positive perception of social self-efficacy will reduce depressive symptoms. Social interaction plays an important role as children enter into adolescents. Self-efficacy is also thought to influence depression through its impact on social relationships. Previous studies suggest doubting one’s ability to interact and connect with other people makes it more difficult to form the types of positive social relationships that make one less vulnerable to depression (47). Social self-efficacy has also been found to predict greater depressive symptoms in both adults (48) and children (42).

Adolescence can be a difficult time for students and for those close to them due to the transitional changes and environmental pressures. Self-efficacy contributes as motivational source, helps to be consistent and put in a more efforts to cope with the demands related to transitional changes. While, beliefs of in-competency related to interpersonal relationships lead to shyness, loneliness, poor self-esteem, and fears in social interaction. These constructs may result in self-debilitating thoughts, cause depressive symptom and also exacerbate the severity of depression. This is validated by longitudinal research conducted to explore the impact of self-efficacy beliefs on self-reported tendencies to experience shyness in interpersonal encounters among a population of adolescents. Findings indicated that two of the four self-efficacy measures (forming and maintaining social relationships, dealing effectively with parents, managing negative emotions, and expressing positive emotions toward others) uniquely contributed to levels of shyness and that perception of social self-efficacy uniquely contributed to shyness (49). Similarly, Bandura (11) also proposed that individuals with low level of self-efficacy appear as shy, try to escape from challenges, are quick to discontinue complex processes, and are prone to higher levels of depression and stress. As fears in social situations, lack of assertiveness and shyness lead the individual to be isolated and alone (50). Galanaki and Kalantzi-Azizi (51) have found that self-efficacy for peer interaction is related to both loneliness and social dissatisfaction. According to the longitudinal study, social self-efficacy mediated the association between attachment anxiety and feelings of loneliness and subsequent depression, whereas self-disclosure mediated the association between attachment avoidance and feelings of loneliness and subsequent depression (52).

The beliefs people hold about their efficacy influence over the events that affect their life and shape the personal development. These beliefs serve as a regulatory function in all spheres of life. A large number of researches indicate that doubt over the capabilities to control the situation leads toward hopelessness, depressive symptoms, and suicidal ideation (53-55). Whereas adolescents’ self-efficacy beliefs to manage positive and negative emotions and interpersonal relationships contribute to promote positive expectations about the future, to maintain a high self-concept, to perceive a sense of satisfaction for life and to experience more positive emotions (56). Mazza and Reynolds (57) conducted the longitudinal study to investigate the relationship of psychological and social-environmental factor with adolescent suicidal ideation in student. Findings indicate that daily hassles and negative life event for male and social support and depression for female were a significant factor related to suicidal ideation. Another research finding indicates that respondents with prior depression, dependent life events had a significant negative impact on self-efficacy (58).

Poor relationship qualities in close relationships and low PSSE might be associated with adolescents’ depressive symptoms and prevalence of depression. McFarlane et al. (59) found that social self-efficacy and social support from family and peers are interrelated in their links with depression. Researches have also shown that those college students who feel loneliness have the possibility to become depressed and loneliness was positively associated with depression (60, 61). Lonely people have been judged to be less competent in interpersonal relationships then people who are not feeling alone (62, 63). Jenkins et al. (64) also confirmed that perceived low parental intimate support, high conflict with parents, and lower perceived self-efficacy were related to depressive symptoms.

On the contrary, adolescents’ efficacious beliefs about the ability to express, and share positive emotions with others, help them building satisfying interpersonal relationships constitute the main sources of positive emotional experiences. People who have good interpersonal and social skills are able to play an efficient role in social situations. Shu-Ping (65) found that higher level of social self-efficacy also predicts a lower level of acculturation stress. Positive and high efficacious beliefs of individuals are considered an important personal resource in adjustment to new demands of life and circumstances. According to transactional stress theory (66), psychological adaptation to new circumstances is influenced by psychological resources, such as the sense of perceived efficacy to master new environmental demands. Ideally, in this period adolescent’s sense of self-efficacy for being able to exert a good measure of control over their lives, or agency (67) plays a pivotal role in subjective well-being and subjective sense of purpose in life (68).

## Conclusion

These findings strongly support the existing literature that positive emotions and thinking patterns may help the people in good adaptation and healthier human functioning. Adolescents’ psychological well-being is also influenced, by their beliefs to manage successfully their positive and negative emotional experiences in various life events and transitional changes. 

## Authors' contributions

ZR conceived and designed the evaluation, helped to draft the manuscript and interpret the clinical data. SY participated in designing the evaluation, collected the clinical data and revised the manuscript. RA re-evaluated the clinical data, performed the statistical analysis and revised the manuscript. All authors read and approved the final manuscript.

## Notes

**Declaration of interest:** None.
